# Excessive Inflammatory Response to Bacterial Lymphadenitis in an Infant With A Family History of Recurrent Lymph Node Swelling: A Case Report

**DOI:** 10.7759/cureus.104832

**Published:** 2026-03-07

**Authors:** Mariko Inaba, Yosuke Baba, Kaho Nakamura, Shun Toriumi, Hiromichi Shoji

**Affiliations:** 1 Department of Pediatrics, Juntendo University Shizuoka Hospital, Shizuoka, JPN; 2 Department of Pediatrics, Juntendo University Faculty of Medicine, Tokyo, JPN

**Keywords:** family history, hemophagocytic lymphohistiocytosis (hlh), infant, inflammation and inflammatory markers, lrba gene, suppurative lymphadenitis

## Abstract

Suppurative lymphadenitis in infancy typically responds well to antimicrobial therapy. However, some cases exhibit elevated inflammatory markers relative to clinical severity, raising questions about host inflammatory regulation. We report a one-month-old female infant with suppurative lymphadenitis caused by methicillin-sensitive *Staphylococcus aureus* (MSSA). Despite an appropriate clinical response to treatment, she demonstrated elevated ferritin (446 ng/mL) and soluble IL-2 receptor (1,734 U/mL). Systematic evaluation excluded hemophagocytic lymphohistiocytosis and primary immunodeficiency. Family history revealed recurrent lymph node swelling in the maternal lineage. Exploratory genetic testing identified a heterozygous *LRBA* gene variant (c.8417A>T), classified as a variant of uncertain significance. This case illustrates the importance of systematic evaluation when inflammatory markers appear elevated relative to clinical presentation. The identified *LRBA* variant is of uncertain significance and does not establish causality. Long-term follow-up is warranted to clarify whether this represents a clinically meaningful phenotype.

## Introduction

Suppurative lymphadenitis in infancy is a relatively common condition in clinical practice, most frequently caused by methicillin-sensitive *Staphylococcus aureus* (MSSA) [1]. The majority of cases respond favorably to appropriate antimicrobial therapy and, when necessary, surgical drainage. However, clinicians occasionally encounter cases where inflammatory markers appear elevated relative to clinical severity, prompting consideration of underlying host factors.

Ferritin and soluble interleukin-2 receptor (sIL-2R) are acute-phase reactants that increase during infection and inflammation. Marked elevations, particularly when disproportionate to the degree of infection, can indicate conditions such as hemophagocytic lymphohistiocytosis (HLH) or other immune dysregulation syndromes [2]. Host factors, including genetic variants affecting immune regulation, may influence the magnitude of inflammatory responses to infection [3]. LRBA (lipopolysaccharide-responsive beige-like anchor protein) is a regulator of CTLA-4 expression on T cells; biallelic loss-of-function mutations cause autosomal recessive immune dysregulation [4].

We report a one-month-old infant with MSSA suppurative lymphadenitis who exhibited inflammatory markers that appeared elevated relative to her clinical presentation. Notable features included a family history of recurrent lymph node swelling and identification of a heterozygous *LRBA* gene variant of uncertain significance (VUS). This case emphasizes the importance of systematic evaluation in such presentations.

## Case presentation

A one-month and 22-day-old Japanese female infant presented to our emergency department with left auricular swelling and fever. She was born at 39 weeks and three days of gestation via normal vaginal delivery without perinatal complications. Birth weight was 3,120 g. The neonatal period was uneventful. At her one-month health check-up, infantile eczema was noted, and an emollient was prescribed. She had no history of severe infections and was not on any medications. No vaccinations had been administered due to her young age.

Family history revealed notable findings in the maternal lineage. Both the mother and maternal grandmother had a history of recurrent lymph node swelling. The mother experienced recurrent cervical lymph node enlargement 2-3 times per year during childhood, sometimes accompanied by fever, all of which resolved spontaneously without requiring investigation. Similar episodes were reported in the maternal grandmother, although details were limited. The patient was the first child with no siblings.

One day prior to the presentation (day of life 51), the patient developed a palpable mass behind the left auricle with mild erythema. The following day, multiple masses became palpable with progressive swelling, and purulent discharge appeared on the skin surface. She was initially evaluated at a local clinic and then referred to our hospital due to fever (38.7°C) and suspected suppurative lymphadenitis. Despite these findings, oral intake was relatively maintained, and overall condition remained stable.

Physical examination on admission revealed a well-appearing infant with a body weight of 4,320 g (growth appropriate), a body temperature of 38.5°C, a heart rate of 156 beats per minute, a respiratory rate of 42 breaths per minute, and an oxygen saturation of 98% on room air. The infant was alert and active. Respiratory sounds were clear, heart sounds were regular without murmurs, and the abdomen was soft and flat without hepatosplenomegaly. No rash or joint swelling was observed. The local examination revealed multiple tender, soft nodules measuring 6×8 mm posterior to the left auricle extending to the neck, 10×20 mm in the left preauricular region, and 10×10 mm on the posterior auricular surface. All lesions showed erythema and tenderness, with the infant crying upon palpation, and purulent discharge from the skin surface, suggesting an infectious etiology. No external malformations or accessory auricles were noted.

Laboratory findings on admission are summarized in Table 1. The white blood cell count was elevated at 18,300/μL with neutrophilic predominance, while hemoglobin and platelet counts were within normal limits. C-reactive protein was elevated at 4.65 mg/dL. Notably, ferritin was 446 ng/mL, and the soluble interleukin-2 receptor (sIL-2R) was 1,734 U/mL. While reference ranges vary by age and assay, ferritin levels in healthy one- to two-month-old infants typically range from 50 to 200 ng/mL, and sIL-2R is generally <500 U/mL in pediatric populations [5,6]. Triglycerides (112 mg/dL) and fibrinogen (298 mg/dL) were within normal ranges. Blood, urine, and cerebrospinal fluid cultures all remained negative.

**Table 1:** Laboratory Findings on Admission Inflammatory markers were elevated, with ferritin exceeding twice the upper limit of normal for age and soluble IL-2 receptor (sIL-2R) exceeding three times the pediatric reference range. These values appeared disproportionate to those expected for a localized MSSA infection in this age group. MSSA, methicillin-sensitive Staphylococcus aureus; IL-2, interleukin-2; IgG, immunoglobulin G; IgA, immunoglobulin A; IgM, immunoglobulin M; NBT, nitroblue tetrazolium; CH50, 50% hemolytic complement.

Parameter	Value	Reference Range
Complete Blood Count		
White blood cell count (/μL)	18,300	6,000–17,500
Neutrophils (%)	72	20–70
Lymphocytes (%)	20	20–70
Monocytes (%)	8	1–11
Hemoglobin (g/dL)	11.2	10.0–14.0
Platelet count (×10³/μL)	385	150–450
Blood Chemistry		
AST (U/L)	28	10–40
ALT (U/L)	16	5–35
LDH (U/L)	312	200–400
Triglycerides (mg/dL)	112	<200
Fibrinogen (mg/dL)	298	170–410
Inflammatory Markers		
CRP (mg/dL)	4.65	<0.30
Ferritin (ng/mL)	446	<100 (age-adjusted)
Soluble IL-2 receptor (U/mL)	1,734	<500 (pediatric)
Immunological Evaluation		
IgG (mg/dL)	456	250–550
IgA (mg/dL)	8	5–20
IgM (mg/dL)	32	20–80
CD3+ T cells (%)	68	55–75
CD4+ T cells (%)	47	35–55
CD8+ T cells (%)	19	15–25
CD19+ B cells (%)	22	15–30
NK cells CD16+56 (%)	9	5–15
NBT reduction test (%)	94	≥90
CH50 (U/mL)	42	30–50
C3 (mg/dL)	89	65–135
C4 (mg/dL)	18	13–35
Microbiology		
Blood culture	Negative	-
Urine culture	Negative	-
CSF culture	Negative	-
Wound culture (abscess)	MSSA	-

**Figure 1:**
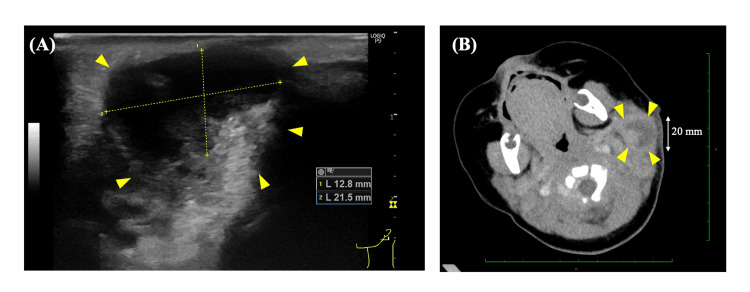
Imaging Studies of Suppurative Lymphadenitis Panel A: Cervical ultrasonography demonstrating an enlarged lymph node in the left preauricular region measuring 12.8 mm × 21.5 mm. The lymph node shows hypoechoic areas (yellow arrowheads) suggestive of liquefaction and abscess formation. Yellow measurement calipers indicate the two dimensions of the largest lymph node. Panel B: Contrast-enhanced computed tomography (CT) of the neck in axial view, showing multiple enlarged lymph nodes in the left parotid region with central low-density areas (yellow arrowheads) consistent with abscess formation. The scale bar indicates 20 mm. No airway compromise or extension into deeper neck spaces is evident. These images were obtained at Juntendo University Shizuoka Hospital as part of routine clinical care. Written informed consent for publication of these anonymized images was obtained from the patient's parents. All identifiable information has been removed to protect patient privacy. CT, computed tomography.

Imaging studies were performed to characterize the extent of lymph node involvement and to assess for complications. Cervical ultrasonography revealed multiple enlarged lymph nodes in the left preauricular, mastoid, and posterior auricular regions, as shown in Figure 1A. The largest lymph node measured 21.5 mm in maximum diameter and demonstrated hypoechoic areas suggestive of liquefaction, consistent with abscess formation. Contrast-enhanced computed tomography of the neck confirmed these findings and provided additional anatomical detail (Figure 1B). Multiple lymph nodes with central low-density areas were identified in the left parotid region, consistent with suppurative lymphadenitis with abscess formation. The airway was patent without evidence of compromise, and there was no extension of infection into deeper neck spaces or the mediastinum.

Given the elevated inflammatory markers, we systematically evaluated potential underlying conditions. The differential diagnosis included HLH, primary immunodeficiency disorders, and autoinflammatory syndromes. HLH was considered, given the fever and elevated ferritin and sIL-2R, although the patient lacked splenomegaly, cytopenias, and other typical features.

We systematically evaluated the patient against the HLH-2004 diagnostic criteria, which require at least five of eight criteria for diagnosis [7]. Of the eight criteria, only fever ≥38.5°C was present. Splenomegaly was absent. Cytopenias affecting two or more cell lineages were not observed, as white blood cell count was elevated and both hemoglobin and platelet count were within normal limits. Hypertriglyceridemia and hypofibrinogenemia were both absent. Hemophagocytosis was not assessed, as clinical suspicion was low. Natural killer cell activity was not measured. The ferritin level was 446 ng/mL, below the HLH threshold of 500 ng/mL. Soluble CD25 (sIL-2R) was 1,734 U/mL, below the diagnostic threshold of 2,400 U/mL. Only one of eight criteria was fulfilled; HLH was considered unlikely.

Comprehensive immunological evaluation revealed normal immunoglobulin levels, normal lymphocyte subsets, normal neutrophil function (NBT reduction test 94%), and normal complement levels. These studies excluded primary immunodeficiency.

**Figure 2:**
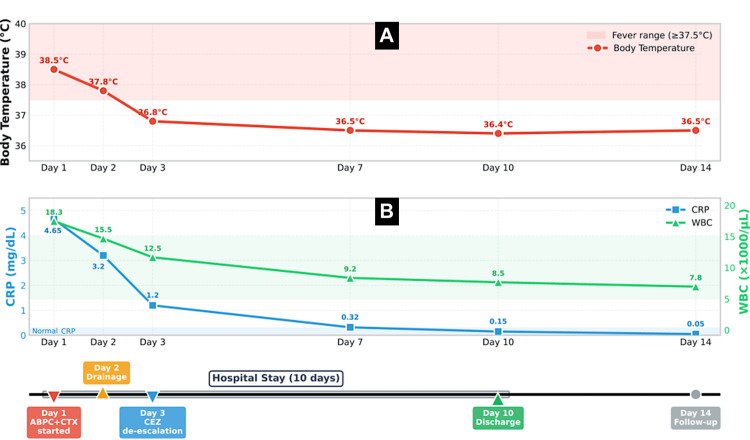
Clinical Course of Body Temperature and Inflammatory Markers The graph illustrates the temporal changes in (A) body temperature (red line) and (B) C-reactive protein (blue line) and white blood cell count (green line) throughout the clinical course. Day 1 marks hospital admission and initiation of empiric antibiotic therapy with ampicillin and cefotaxime (ABPC+CTX). On Day 2, needle aspiration and drainage were performed. On Day 3, antibiotic therapy was de-escalated to cefazolin (CEZ) following identification of methicillin-sensitive *Staphylococcus aureus*. Rapid defervescence was observed following appropriate antimicrobial therapy. By Day 7, a significant improvement in inflammatory markers was evident. The patient was discharged on Day 10. At the two-week follow-up visit (Day 14), complete normalization of inflammatory markers was confirmed. The pink-shaded area indicates the fever range (≥37.5°C). This figure was created by the authors using the Python Matplotlib library (NumFOCUS, Austin, Texas, USA). CRP, C-reactive protein; WBC, white blood cell count; ABPC, ampicillin; CTX, cefotaxime; CEZ, cefazolin.

We initiated empiric therapy with ampicillin and cefotaxime intravenously. The temporal course of body temperature and inflammatory markers is illustrated in Figure 2. On hospital day 2, needle aspiration and drainage were performed. Culture from the abscess cavity grew MSSA susceptible to cefazolin. Blood cultures remained negative. Antimicrobial therapy was de-escalated to intravenous cefazolin on hospital day 3. As shown in Figure 2, the patient defervesced promptly, with body temperature declining to 36.8°C on hospital day 3. By hospital day 7, inflammatory markers improved significantly (CRP 0.32 mg/dL, WBC 9,200/μL). The patient was discharged on day 10. At the two-week follow-up, inflammatory markers had normalized (CRP <0.10 mg/dL, WBC 7,800/μL, ferritin 98 ng/mL) with complete resolution of lymphadenopathy.

Given the family history of recurrent lymph node swelling, we conducted exploratory genetic testing using a next-generation sequencing panel targeting immune-related genes. A heterozygous missense variant in the *LRBA* gene was identified: c.8417A>T (p.Lys2806Met), classified as a VUS according to ACMG criteria [8]. The variant has an allele frequency of 0.000013 in the gnomAD database [9], is not registered in ClinVar, and in silico predictions suggest benign or tolerated effects. *LRBA* deficiency is an autosomal recessive disorder; heterozygous carriers are typically asymptomatic [10]. Our patient does not exhibit the clinical phenotype of *LRBA* deficiency. The pathogenic significance of this variant remains uncertain.

## Discussion

This case demonstrates a one-month-old infant with MSSA suppurative lymphadenitis who exhibited inflammatory markers that appeared elevated relative to her clinical presentation, systematic exclusion of HLH and primary immunodeficiency, a family history of recurrent lymph node swelling, and identification of an *LRBA* gene VUS.

Interpretation of inflammatory markers

While ferritin and sIL-2R are acute-phase reactants that increase during bacterial infections, the observed levels (ferritin 446 ng/mL, sIL-2R 1,734 U/mL) appeared elevated given the localized nature of the infection and the patient's rapid clinical response. However, we acknowledge important limitations in this interpretation. First, age-specific reference data for ferritin and sIL-2R in infants with uncomplicated bacterial infections are limited in the literature. Second, substantial biological variability exists among individuals. Third, the elevated values may simply reflect the upper end of normal inflammatory responses rather than a pathological process. Nevertheless, these findings prompted a systematic evaluation, which appropriately excluded more serious conditions such as HLH.

Family history and genetic findings

The recurrent lymph node swelling in the mother and maternal grandmother is intriguing but non-specific. Without detailed medical records or examination of affected relatives, the clinical significance remains uncertain. The identified *LRBA* variant is classified as VUS and should not be interpreted as pathogenic. *LRBA* deficiency follows autosomal recessive inheritance; heterozygous carriers typically remain asymptomatic [10,11]. Our patient lacks the characteristic clinical features of *LRBA* deficiency, including autoimmune enteropathy, hypogammaglobulinemia, and severe recurrent infections. Critically, we did not perform functional studies of *LRBA* expression or CTLA-4 function, nor did we conduct family segregation analysis to determine whether the variant tracks with the inflammatory phenotype. The variant should be considered a genetic finding of uncertain clinical significance rather than an explanation for the observed phenotype.

Hypothetical mechanistic considerations

**Figure 3:**
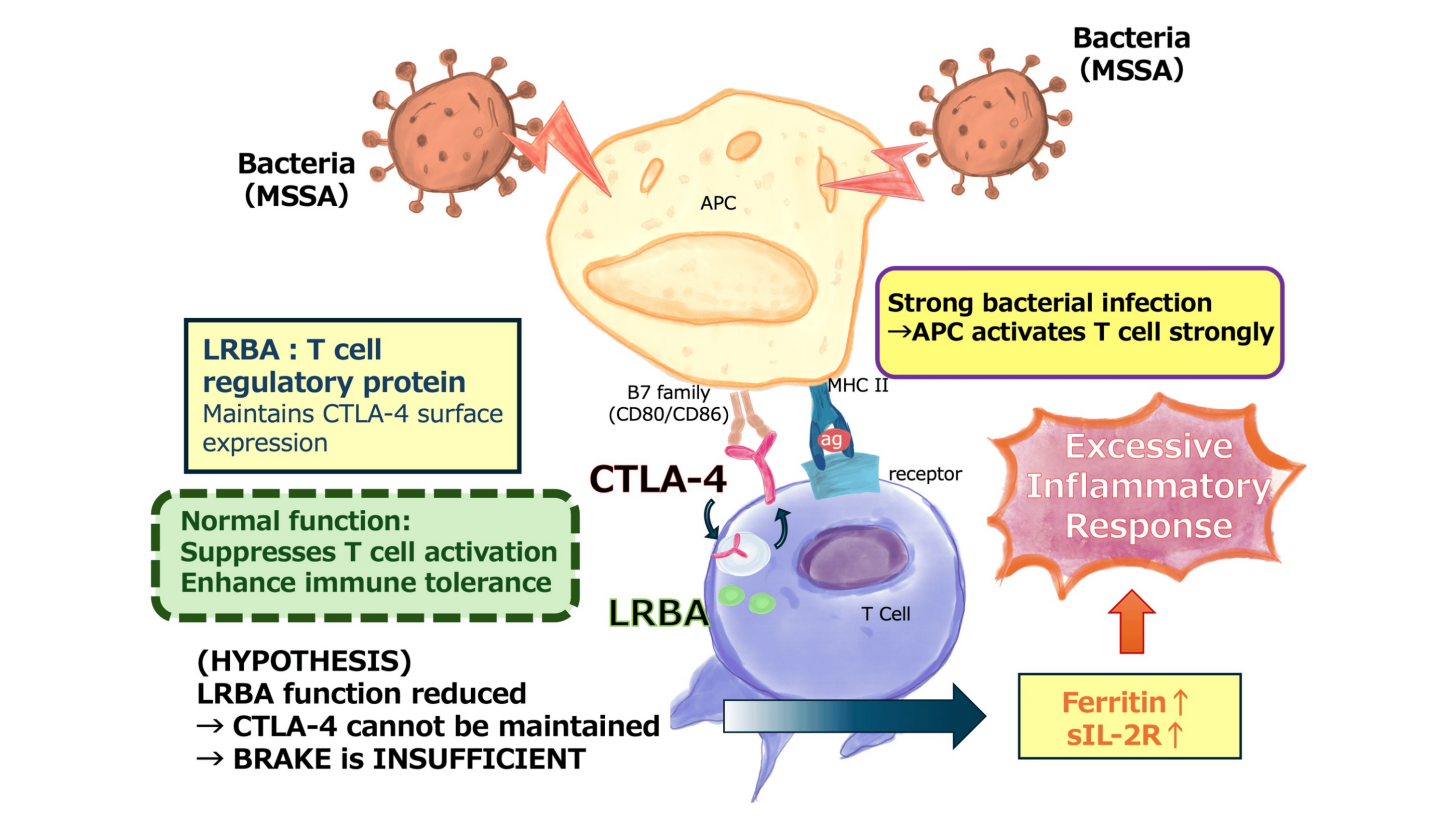
Hypothetical Mechanism of Excessive Inflammatory Response Through the LRBA-CTLA-4 Pathway Schematic illustration depicting the potential mechanism underlying the excessive inflammatory response observed in this case. The diagram shows the interaction between bacterial infection (MSSA), antigen-presenting cells (macrophages), and T cell regulation through the *LRBA*-CTLA-4 pathway. Under normal circumstances, *LRBA* maintains cell surface expression of CTLA-4, suppressing T cell activation and maintaining immune homeostasis. In this case, strong bacterial antigen stimulation may have led to excessive T cell activation, potentially exacerbated by suboptimal *LRBA*-CTLA-4 function, resulting in disproportionately elevated inflammatory markers (ferritin↑, sIL-2R↑) despite adequate infection control. Note: This represents a hypothetical mechanism. The pathogenic significance of the identified *LRBA* variant remains uncertain, and functional studies would be required to establish causality. This figure was created by the authors. The diagram represents original work by the authors based on established biological pathways described in the referenced literature. LRBA, lipopolysaccharide-responsive beige-like anchor protein; CTLA-4, cytotoxic T-lymphocyte-associated protein 4; MSSA, methicillin-sensitive Staphylococcus aureus; sIL-2R, soluble interleukin-2 receptor.

Figure 3 presents a hypothetical model by which the *LRBA*-CTLA-4 pathway dysfunction could theoretically contribute to excessive inflammatory responses. This model is based on established biological mechanisms whereby *LRBA* maintains CTLA-4 surface expression on T cells, and CTLA-4 negatively regulates T cell activation [10,11]. However, this remains purely speculative in the context of our patient. We emphasize that this model represents a hypothesis rather than a demonstrated mechanism. The heterozygous *LRBA* variant has not been functionally validated, its pathogenic significance is uncertain, and alternative explanations must be considered, including normal biological variability, strain-specific bacterial factors, or other unidentified host factors. Functional studies would be required to establish any causal relationship.

Clinical implications

When inflammatory markers appear elevated relative to clinical presentation in pediatric infectious diseases, systematic evaluation for conditions such as HLH and primary immunodeficiency is warranted. Detailed family history may provide diagnostic clues. However, genetic variants of uncertain significance should be interpreted cautiously and do not guide clinical management. In this case, the *LRBA* variant does not establish causality and should not influence treatment decisions.

Limitations

This report has significant limitations. We lack comparative data defining typical ferritin and sIL-2R levels in age-matched infants with uncomplicated MSSA infections. We did not perform functional validation of the *LRBA* variant, family segregation analysis, or assessment of CTLA-4 expression or T cell function. The clinical follow-up period is short. Detailed characterization of the family phenotype was not possible. These limitations preclude definitive conclusions about the pathogenic significance of the identified variant or its relationship to the observed inflammatory markers.

## Conclusions

We report a one-month-old infant with MSSA suppurative lymphadenitis who exhibited inflammatory markers that appeared elevated relative to clinical presentation, with systematic exclusion of HLH and primary immunodeficiency. A heterozygous *LRBA* gene VUS was identified but does not establish causality or guide management. This case emphasizes the importance of systematic evaluation when inflammatory responses appear atypical. Long-term clinical follow-up is recommended to monitor for recurrent infections or immune manifestations. Functional studies and family segregation analysis would be needed to clarify the significance of the genetic finding.
